# Influence of advancing age on clinical presentation, treatment efficacy and safety, and long-term outcome of inducible paroxysmal supraventricular tachycardia without pre-excitation syndromes: A cohort study of 1960 patients included over 25 years

**DOI:** 10.1371/journal.pone.0187895

**Published:** 2018-01-05

**Authors:** Béatrice Brembilla-Perrot, Jean Marc Sellal, Arnaud Olivier, Thibaut Villemin, Daniel Beurrier, Julie Vincent, Vladimir Manenti, Christian de Chillou, Erwan Bozec, Nicolas Girerd

**Affiliations:** 1 Department of Cardiology, Nancy University Hospital, Vandoeuvre-lès-Nancy, France; 2 INSERM, Centre d’Investigations Cliniques 1433, Nancy, France; 3 INSERM, Unité 1116, Nancy, France; 4 Faculté de médecine, Université de Lorraine, Nancy, France; 5 CHU de Nancy, Institut Lorrain du Cœur et des Vaisseaux, Nancy, France; 6 INI-CRCT (Cardiovascular and Renal Clinical Trialists) F-CRIN network, Nancy, France; Universita degli Studi di Roma La Sapienza, ITALY

## Abstract

**Aim:**

To investigate the influence of increasing age on clinical presentation, treatment and long-term outcome in patients with inducible paroxysmal supraventricular tachycardia (SVT) without pre-excitation syndromes.

**Methods:**

Clinical and electrophysiological study (EPS) data, as well as long-term clinical outcome (mean follow-up 2.4±4.0 years) were collected in patients referred for regular tachycardia with inducible SVT during EPS without pre-excitation.

**Results:**

Among 1960 referred patients, 301 patients (15.4%) were aged ≥70 (70–97). In this subset, anticoagulants were prescribed in 49 patients following an erroneous diagnosis of atrial tachycardia and 14 were previously erroneously diagnosed with ventricular tachycardia because of wide QRS. Ablation was performed more frequently in patients ≥70 despite more frequent failure and complications. During follow-up, higher risks of AF, stroke, pacemaker implantation and death were observed in patients ≥70 whereas SVT recurrences were similar in both age groups. In multivariable analysis, age ≥70 was independently associated with higher risks of SVT-related adverse events prior to ablation (OR = 1.93, 1.41–2.62, p<0.001), conduction disturbances (OR = 11.27, 5.89–21.50, p<0.001), history of AF (OR = 2.18, 1.22–3.90, p = 0.009) and erroneous diagnosis at baseline (OR = 9.14, 5.93–14.09, p<0.001) as well as high rates of procedural complications (OR = 2.13, 1.19–3.81, p = 0.01) and ablation failure (OR = 1.68, 1.08–2.62, p = 0.02). In contrast, age ≥70 was not significantly associated with a higher risk of AF in multivariable analysis.

**Conclusions:**

A sizeable proportion of patients with inducible SVT without pre-excitation syndromes are elderly. These patients exhibit higher risks of erroneous tachycardia diagnosis prior to EPS as well as failure and/or complication of ablation, but similar risk of SVT recurrence. These results support performing transesophageal EPS in most patients and intracardiac EPS in selected patients. EPS may furthermore prove useful in elderly patients with regular tachycardia, mainly by avoiding treatment based on an erroneous diagnosis.

## Introduction

Atrial fibrillation (AF) and atrial tachycardia (AT) are common arrhythmic conditions in elderly persons and are associated with a higher risk of embolic stroke [1]. Atypical atrial flutter and macroreentrant AT are on the rise as consequences of increasing AF ablation in older populations, yet there are limited outcome data reported for this segment of the population [1]. While anticoagulants are indicated in atrial flutter [2,3], regular supraventricular tachycardias (SVT) without evidence of atrial flutter are frequently treated as AT with anticoagulants, which is not straightforwardly recommended by guidelines [2,3], although such treatment is in line with certain expert reports [4].

Paroxysmal SVT is a common cause of regular tachycardia [5]. SVT is considered as benign and ablation is only required in symptomatic patients. Age-related differences have been reported for SVT [6–8] with a higher prevalence of structural heart disease and longer AH intervals at baseline. However, data on the long-term follow-up of SVT without pre-excitation syndrome in elderly patients remain limited and the possible benefit of etiological evaluation of regular tachycardia in these elderly patients has been insufficiently reported.

The diagnosis of SVT is often made in the emergency department, although it is common to elicit symptoms suggestive of SVT before initial electrocardiogram/electrocardiographic (ECG) documentation. The current ACC/AHA/HRS guidelines state that “EP study with the option of ablation is useful for the diagnosis and potential treatment of SVT” [4]. However, they do not specify the diagnostic algorithm that should be used in patients with suspected SVT (i.e. without definitive evidence of a SVT). In addition, with regard to the specific management of SVT in elderly patients, these guidelines state that “Diagnostic and therapeutic approaches to SVT should be individualized in patients more than 75 years of age to incorporate age, comorbid illness, physical and cognitive functions, patient preferences, and severity of symptoms”. Yet, much of the evidence leading to this recommendation is based on therapeutic data rather than evidence centered on the diagnosis of SVT. As a result, the place of electrophysiological study (EPS) in older patients is currently insufficiently evidence-based.

In light of the above and given the absence of large-scale data, the present study aimed to assess the influence of age on clinical presentation, treatment and long-term outcome of **inducible paroxysmal SVT without pre-excitation syndromes** in a large cohort of patients.

## Methods

### Study population

**Fig 1:**
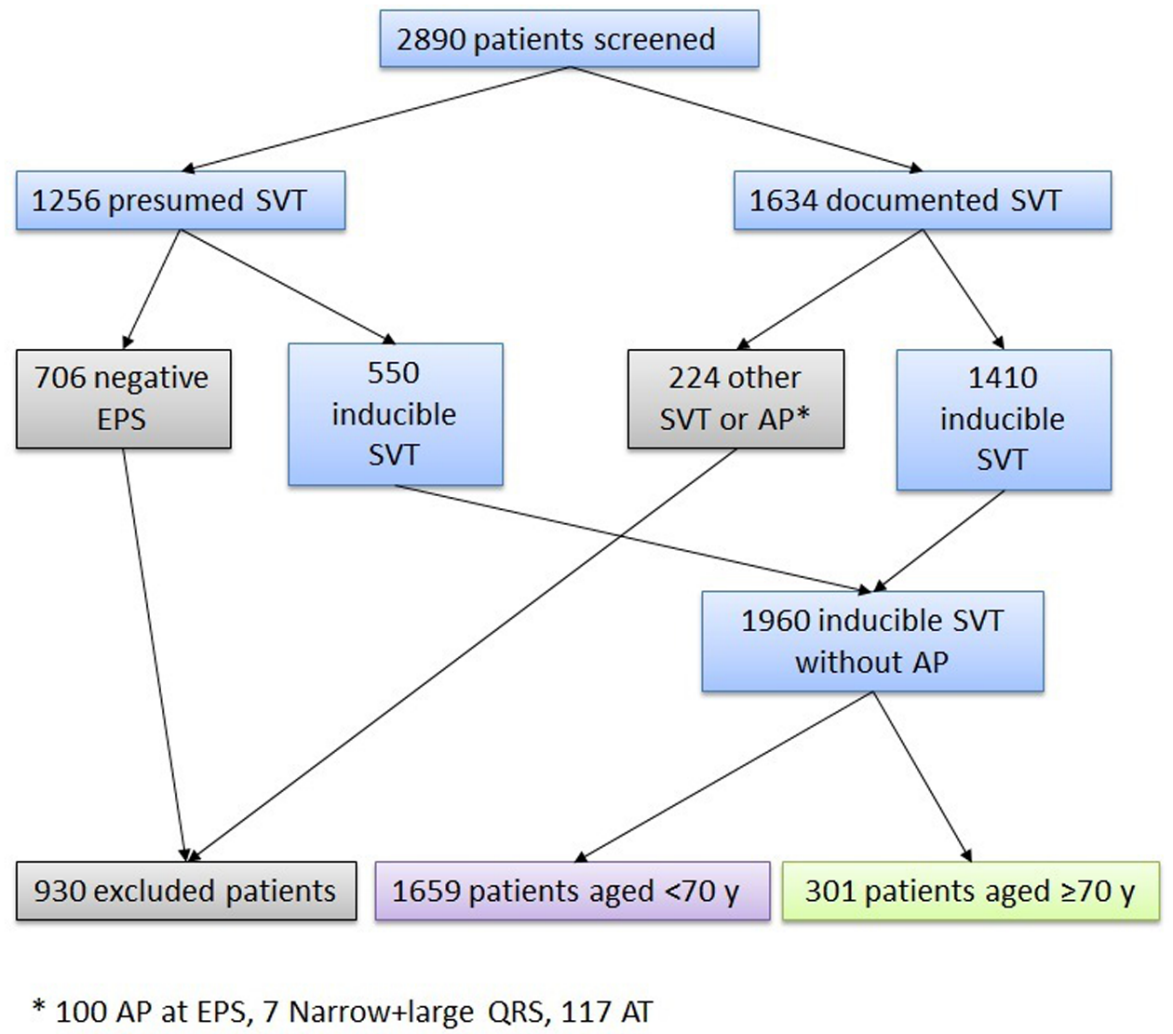
Flow chart illustrating the recruitment process. SVT: supraventricular tachycardia, AP: anterograde conduction over an accessory pathway only visible at atrial pacing, EPS: electrophysiological study, AT: atrial tachycardia, VT: ventricular tachycardia.

The present is a retrospective observational study of 2890 patients consecutively referred for a presumed (n = 1256) or documented (n = 1634) regular paroxysmal SVT without pre-excitation syndrome or antidromic AV reciprocating tachycardia (AVRT) due to anterograde conduction through an accessory pathway in a tertiary care center between January 1990 and October 2015. Patients in whom atrial fibrillation or atrial flutter was suspected to be the cause of symptoms were not considered in this analysis. Patients with symptoms of regular tachycardia that were not attributed to atrial flutter were referred from non-interventional hospitals or cardiology practices in the area of our hospital. Patients were usually referred directly in our center for a short hospital stay, after the approval of one of our EP physicians, in order to undergo a systematic workup for SVT. However, in some patients, the first encounter with an EP physician occurred in the outpatient clinic. The EP physician then decided whether the aforementioned short hospital stay was needed to document and/or treat the arrhythmia. Patients without inducible SVT during the work-up (706 of 1256 patients with presumed SVT), or with either anterograde conduction over an accessory pathway appearing at atrial pacing, ventricular tachycardia or atrial tachycardia (224 of 1634 patients with documented tachycardia) were excluded as depicted in the flowchart illustrating the recruitment process (Fig 1). As a result, 1960 patients were available for the analysis. Of note, the treating cardiologists, who discussed this diagnosis with an electrophysiologist prior to referring the patient, made the initial diagnosis of SVT.

### Data extraction

As in our previous studies [9–11], clinical data were retrospectively extracted from the patients’ medical records. The study was approved by the ***Commission nationale de l'informatique et des libertés*** *(CNIL)*. Under French law, no formal IRB approval is required for data extraction from patients’ medical records.

The following data were collected: age, gender, the presence of associated heart disease, history of AF, history of stroke, presence of diabetes, presence of conduction disturbances and occurrence of adverse events. Adverse events that occurred prior to or during the work-up were classified as major (requiring resuscitation) or minor (events that required patient hospitalization). These included syncope, ischemic coronary event, acute heart failure or other poorly-tolerated event directly related to a SVT episode.

### Electrophysiology laboratory protocol

All patients underwent an electrophysiological study as part of the systematic work-up performed for SVT. Electrophysiological studies were performed after signing the clinical informed consent form endorsed by the French Society of Cardiology (*Societé Française de Cardiologie)*. All antiarrhythmic drugs were discontinued at least five half-lives prior to the study. Electrophysiological study was performed by transesophageal route in patients with undocumented tachycardia (defined as the absence of ECG of the symptomatic event, n = 1256) or in patients with documented tachycardia but of uncertain mechanism (n = 102). Indications for esophageal study were similar in patients aged <70 years (47.5%) and ≥70 years (46.9%) with documented tachycardia and was performed by intracardiac route in view of ablation. Details of the protocol have previously been described [8,10–13]

Briefly, atrial pacing and programmed atrial stimulation were systematically performed during sinus rhythm with atrial pacing conducted at two cycle lengths, 600 and 400 ms. Premature stimuli (S2) were delivered after every eighth paced atrial complex with 10 ms decrements until atrial refractoriness was reached. In the absence of tachycardia induction, isoproterenol (0.02 to 1 μg.min^-1^) was alternatively infused to increase the sinus rate to at least 130 bpm. Atrial pacing was repeated and programmed atrial stimulation was performed at a cycle length of 400 ms.

Diagnosis of SVT was confirmed by electrophysiological study and its mechanism was duly noted.

SVT was classified as typical AV nodal reentry within the atrioventricular node (AVNRT) or atypical AVNRT or reentry within a concealed accessory atrioventricular connection (AVRT).

Atrial vulnerability was defined as the induction of AF or atrial flutter during the protocol.

SVT ablation was performed in symptomatic patients, either immediately after the identification of a SVT mechanism during the electrophysiological study or during a second procedure.

Ablation-related complications were defined as major if they were life-threatening and required the admission of the patient in the intensive care unit or as minor if they regressed without the need of monitoring in intensive care. Complications considered as major were mostly pericardial tamponade requiring emergency drainage, complete AV block requiring pacemaker implantation and death. Complications considered as minor were local bleeding, vagal syncope at femoral puncture, minor pericardial suffusion, transient traumatic or radiofrequency-related second-degree or complete AV block and transient sinus bradycardia.

As a general rule, patients with recurrent tachycardias who refused ablation were discharged with beta-blockers.

### Follow-up

In the case of SVT ablation, patients were discharged without antiarrhythmic medication and were systematically examined by a cardiologist at least one month after ablation as part of usual clinical follow-up.

No treatment was indicated for one or 2 episodes of well-tolerated tachycardia. Medical treatment (initially beta-blockers) was only indicated for recurrent tachycardias when the patient refused the ablation or when the patient was too young for this treatment.

The follow-up was performed by the referring cardiologist and/or general practitioner. This follow-up was available from medical correspondence contained in the patient’s medical record. In addition, information was also collected from patient telephone interviews, as part of the usual clinical follow-up after SVT ablation. General physicians, patients and occasionally other hospitals were contacted to identify clinical outcome. ECG and 24-hour Holter recordings were performed if the patient reported palpitations or symptoms suspected to be the consequence of SVT occurrence/recurrence. Eighty-two patients were lost to follow-up.

The following data were collected: recurrences defined as documented or induced SVT after ablation, false recurrences defined as palpitations but negative on control electrophysiological study, occurrence of AF, stroke, pacemaker implantation and death.

### Statistical methods

Data are expressed as means ± standard deviation (SD) or proportions, as appropriate. Categorical variables were compared using the Chi-square test and continuous variables with the unpaired Student t test.

Multivariable logistic regression was used to assess the association between age and outcomes. All variables associated with a p-value <0.20 on univariable logistic regression analysis were entered in the multivariable models.

To better assess the functional form of the association between age and outcome, a sensitivity analysis was performed using the following age categories: <40, 40–50, 50–60, 60–70 and >70. Given the low number of spontaneous conduction disturbances, this sensitivity analysis could not be performed for this latter outcome.

A p value < 0.05 was considered statistically significant. All statistical analyses were performed using the SPSS package for Windows (version 21, IBM Corp., Armonk, NY, USA).

## Results

### Baseline clinical data

**Fig 2:**
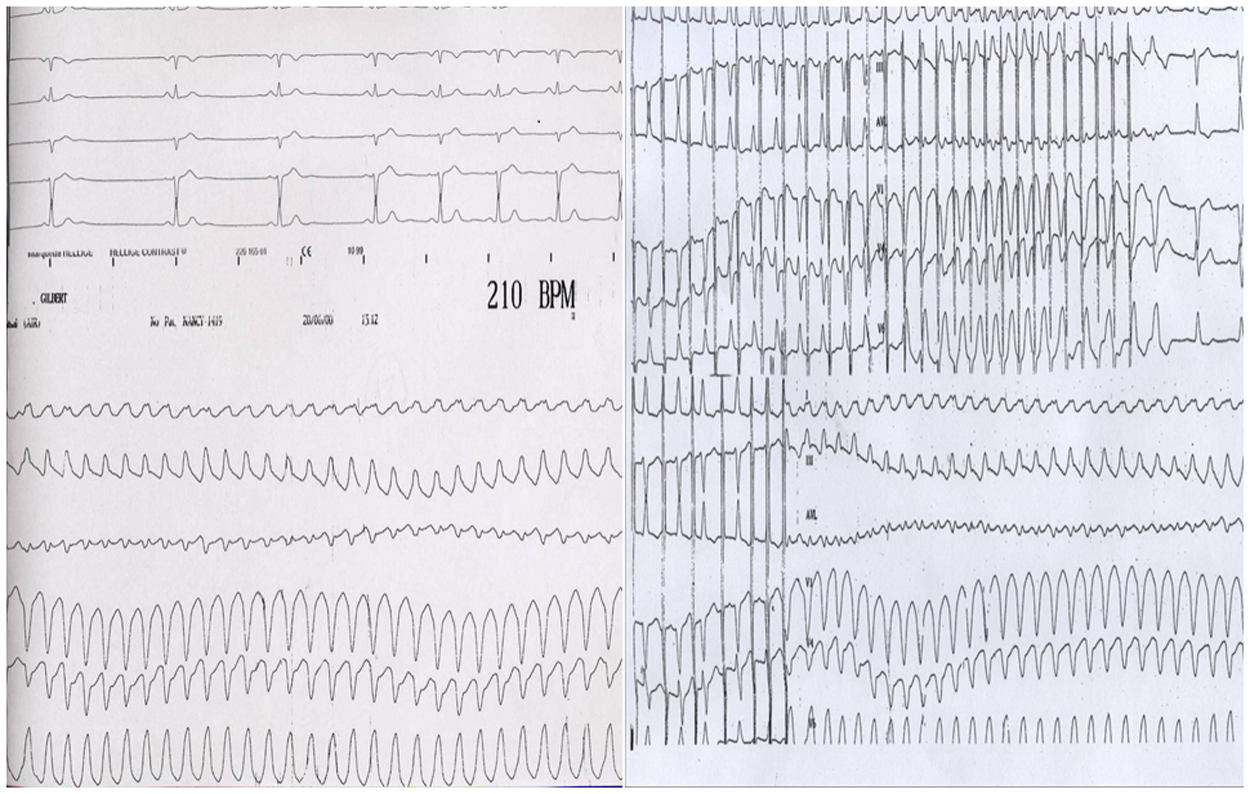
Wide QRS tachycardia in a man aged 85 years and presenting with heart failure. Initial diagnosis of ventricular tachycardia was given in this patient with narrow QRS in sinus rhythm. Spontaneous tachycardia was induced by a premature atrial extrastimulus (esophageal pacing). Ablation of the slow pathway was performed with success in this patient and signs of heart failure disappeared.

Ninety patients ≥70 years were asymptomatic; SVT was noted on Holter monitoring or on emergency ECG for heart failure or angina pectoris. Erroneous diagnosis of atrial tachycardia was frequent in this age group (n = 49, 16.2%, Table 1) often leading to incorrect treatment, mainly anticoagulants and antiarrhytmics. These patients presented regular fine-QRS tachycardia at a rate between 120 and 150 bpm. Elderly patients (n = 14, 4.6%) with confirmed SVT were also frequently diagnosed as having ventricular tachycardia prior to EPS, due to regular wide QRS tachycardia terminating spontaneously or not sensitive to adenosine but suppressed by amiodarone (Fig 2). These diagnoses were made by a cardiologist and generally controlled by an electrophysiologist. In contrast, in patients <70 years, the main diagnostic error was the misdiagnosis of SVT in young women considered as having symptoms attributed to psychological disorders.

**Table 1:** Baseline clinical data in patients ≥70 y and <70 y of age with inducible SVT without pre-excitation syndromes.

	<70 years (N = 1659)	≥70 years (N = 301)	p-value
Age (years)	43±16	77±5	N/A
Female gender	957 (58%)	190 (63%)	0.08
Heart rate (bpm)	191±35	163±30	<0.0001
Heart disease	186 (11.2%)	88 (29.2%)	<0.0001
First-degree AV block	15 (0.9%)	19 (6.3%)	<0.0001
Adverse events	253 (5.2%)	92 (30.6%)	<0.0001
Syncope	224 (13.5%)	57 (19%)	0.01
Adverse events other than syncope	29 (0.2%)	35 (11.6%)	<0.0001
Diabetes	33 (2%)	1 (5.6%)	0.004
AF history	5 (3.2%)	20 (6.6%)	0.004
CHA2DS2-VASc score	0.85±0.73	2.70±0.95	<0.001
Stroke history	32 (2%)	14 (4.6%)	0.004
Erroneous initial diagnosis			
SVT not suspected	83 (5%)	6 (2%)	0.02
Diagnosis of VT	32 (1.9%)	14 (4.6%)	0.004
Diagnosis of AT	10 (0.6%)	49 (16.2%)	<0.0001

SVT: supraventricular tachycardia; AV: atrioventricular; AF: atrial fibrillation; VT: ventricular tachycardia; AT: atrial tachycardia; N/A: not applicable

Patients ≥70 years were more likely to have adverse events related to SVT prior to their EPS and to have heart disease or first-degree AV block (Table 1). Syncope-related SVT was more frequent in elderly patients for which the causes, when identified, differed in patients < 70 and ≥70 years. Associated sinus bradycardia or sinus pauses in sinus rhythm were noted in 14 patients ≥70 years and in only 3 patients < 70 years. Associated conduction disturbances in sinus rhythm were noted in 6 patients ≥70 years and in only 1 patient < 70 years. Vagal reaction (respectively, 20 and 8 patients) and rapid SVT (>220 bpm) (20 and 4 patients) were more frequent in patients < 70 years than in patients ≥70 years. Tetany was only observed before 70 years (n = 6). Heart disease-associated SVT was noted in 8 patients ≥70 years and in only 1 patient < 70 years. In other patients, the clear cause of syncope was not identified.

### Electrophysiological data

AVRT was less frequent in patients ≥70 years than in patients <70 years (Table 2). However, a concealed accessory pathway was identified in 7 women ≥70 years, two of whom were respectively aged 84 and 85 years.

**Table 2:** Electrophysiological data in patients ≥70 y and <70 y of age with inducible SVT without pre-excitation syndromes.

	<70 years (N = 1659)	≥ 70 years (N = 301)	p-value
Typical AVNRT	1170 (70.5%)	234 (77.8%)	0.01
Atypical AVNRT	165 (9.95%)	48 (15.9%)	0.0021
Total AVNRT	1335 (80.5%)	282 (93.7%)	<0.0001
AVRT	324 (19.5%)	19 (6.3%)	<0.0001
Basal induction	1070 (64.5%)	220 (73%)	0.004
Isoproterenol infusion	589 (35.5.2%)	81 (27%)	0.0037
Atrial vulnerability	155 (9.3%)	38 (12.6%)	NS (0.079)
Prolonged AH interval	13 (0.8%)	17 (5.6%)	<0.0001
Prolonged HV interval	2 (0.12%)	4 (1.3%)	0.00048
Associated sick sinus syndrome	3 (0.2%)	6 (2%)	0.00002

AVNRT: Atrioventricular nodal reentry tachycardia; AVRT: atrioventricular reentry tachycardia with concealed accessory pathway; sick sinus syndrome: corrected sinus node recovery time (sinus recovery time—mean sinus cycle length longer than 550 ms)

Associated conduction disturbances such as sinus node dysfunction, suprahisian or infrahisian AV conduction abnormalities were more frequent in patients ≥70 years than in patients <70 years. In patients with an increased AH interval, AVNRT was generally induced after isoproterenol infusion.

### Association between age and outcomes

The mean follow-up duration was 2.8±3 years, with 55% of the population followed for at least one year or more and the remaining patients from one month to one year.

Ablation failure (n = 135) was more frequent in patients ≥70 years than in patients <70 years (Table 3). Causes ranged from the occurrence of irreducible atrial fibrillation to true ablation failure despite correct criteria. Among patients ≥70 years, failure was related to the impossibility (several years ago) of using the retrograde left lateral accessory pathway approach due to severe atherosclerotic lesions in 2 patients ≥70 years and more frequently because of atrial fibrillation in 29 patients. Other causes of ablation failures included the very proximal location of the slow pathway next to the bundle of His (2 cases ≥70 years) and the occurrence of a traumatic complete AV block which only regressed after catheter withdrawal in one patient ≥70 years with a left bundle branch block.

**Table 3:** Ablation procedure data and events during follow-up in patients ≥70 y and <70 y of age with inducible SVT without pre-excitation syndromes.

	<70 years	≥ 70years	p-value
**Ablation procedure data**			
Ablation	1125 (67.8%)	225 (74.8%)	0.0167
Failure of ablation	101 (9.6%)	34 (15%)	0.0028
Complication	49 (4.3%)	18 (8%)	0.021
Major complication	4 (0.35%)	3 (1.3%)	0.062
**Events during follow-up**			
AF during follow-up	83 (5%)	24 (8%)	0.037
Stroke during follow-up	26 (1.6%)	13 (4.3%)	0.0016
Stroke during follow-up in patients without AF history or AF during follow-up	17 (1.1%)	9 (3.4%)	0.004
Recurrence after ablation	83 (7.4%)	13 (5.8%)	0.39
False recurrence	56 (5%)	3 (1.3%)	0.0015
PM implantation	15 (0.9%)	13 (4.3%)	<0.0001
Cardiac death	30 (1.8%)	23 (7.6%)	<0.0001
Follow-up duration	2.97±4.6 years	1.77±17.85	0.0008

AF: atrial fibrillation; PM: pacemaker

Ablation-related complications were more frequent in patients ≥70 years than in patients <70 years, although major complications were not significantly different (Table 3). However, only 2 deaths were directly related to cardiac catheterization and concerned two female patients aged 71 and 88 years, respectively. The first was due to the occurrence of a huge femoral hematoma in a patient with Kahler's disease followed by intravascular disseminated coagulation and consecutive cerebral hemorrhage. In the second case, a cardiovascular collapse occurred when the patient got up the day after the procedure and in whom continuous ECG monitoring did not identify contemporary arrhythmia. The patient had a mild aortic stenosis.

The remaining major events were complete AV block requiring pacemaker implantation in 3 patients <70 years and one patient ≥70 years, and tamponade in a woman aged 61 years.

Later occurrences of AF and stroke were more frequent in patients ≥70 years than in patients <70 years. It should be noted that the rate of AF in patients ≥70 years with initial erroneous diagnosis of atrial tachycardia tended to be lower than the rate of AF in the remaining patients ≥70 years (**1/49, 2% vs. 23/252, 9%) (p = 0.09).**

With regard to stroke, the rate was relatively high compared to the annual incidence of stroke in patients with AF with a CHADS-VASC of 2, which corresponds approximately to a 2% annual incidence. Only one stroke occurred among the 6 patients in whom ablation was performed prior to this event.

### Multivariate analysis of associations with outcomes

Age ≥70 years was independently associated with SVT-related adverse events, spontaneous conduction disturbances, history of AF, erroneous tachycardia diagnosis, complication of ablation and ablation failure. In contrast, age ≥70 was neutrally associated with AF occurrence during follow-up (Table 4).

**Table 4:** Multivariable analysis with SVT-related adverse events, spontaneous conduction disturbances, history of AF, false diagnosis, complications of ablation, ablation failure and AF during follow-up as outcome variables.

Studied outcome	OR	95% Confidence interval		p-value
**SVT-related adverse event**				
Age≥70 y	1.93	1.41	2.62	**<0.001**
Female gender	0.82	0.64	1.05	0.12
Conduction disturbances	1.35	0.65	2.82	0.42
Heart disease	5.28	3.65	7.64	**<0.001**
Diabetes	1.66	0.86	3.18	0.13
**Spontaneous conduction disturbances**				
Age≥70 y	11.27	5.89	21.5	**<0.000**
Female gender	0.34	0.18	0.65	**0.001**
Heart disease	0.11	0.01	0.81	0.03
Diabetes	3.37	1.05	10.85	0.04
**History of AF**				
Age≥70 y	2.18	1.22	3.90	**0.009**
Female gender	0.53	0.33	0.86	**0.01**
History of stroke	1.91	0.56	6.52	0.30
Heart disease	0.50	0.17	1.45	0.20
Diabetes	2.19	0.75	6.43	0.15
**False diagnosis**				
Age≥70 y	9.14	5.93	14.09	**<0.001**
Female gender	0.73	0.48	1.11	0.14
SVT-related adverse event	0.93	0.56	1.54	0.78
Heart disease	2.11	1.30	3.42	**0.003**
History of AF	1.19	0.50	2.86	0.69
**Complications of ablation**				
Age≥70 y	2.13	1.19	3.81	**0.01**
Female gender	1.15	0.69	1.92	0.60
Conduction disturbances	0.48	0.06	3.70	0.49
Heart disease	1.04	0.43	2.53	0.93
Diabetes	1.57	0.47	5.32	0.47
History of AF	1.60	0.56	4.62	0.38
**Failures of ablation**				
Age≥70 y	1.68	1.08	2.62	**0.02**
Female gender	1.32	0.90	1.94	0.16
Accident of ablation	0.75	0.31	1.80	0.53
Conduction disturbances	0.24	0.03	1.80	0.17
Heart disease	2.52	1.37	4.62	**0.003**
Diabetes	1.36	0.54	3.40	0.52
History of AF	2.65	1.37	5.13	**0.004**
**Later occurrence of AF**				
Age≥70 y	1.23	0.72	2.09	0.44
Gender	0.72	0.47	1.10	0.13
History of AF	10.42	5.99	18.16	<0.001
History of stroke	3.40	1.41	8.21	0.007
Heart disease	1.20	0.68	2.12	0.52
Diabetes	1.58	0.58	4.33	0.37
Atrial vulnerability	3.53	2.18	5.71	<0.001
Ablation	1.51	0.90	2.54	0.12

SVT: supraventricular tachycardia; AF: atrial fibrillation

### Sensitivity analysis according to decades of age

**Fig 3:**
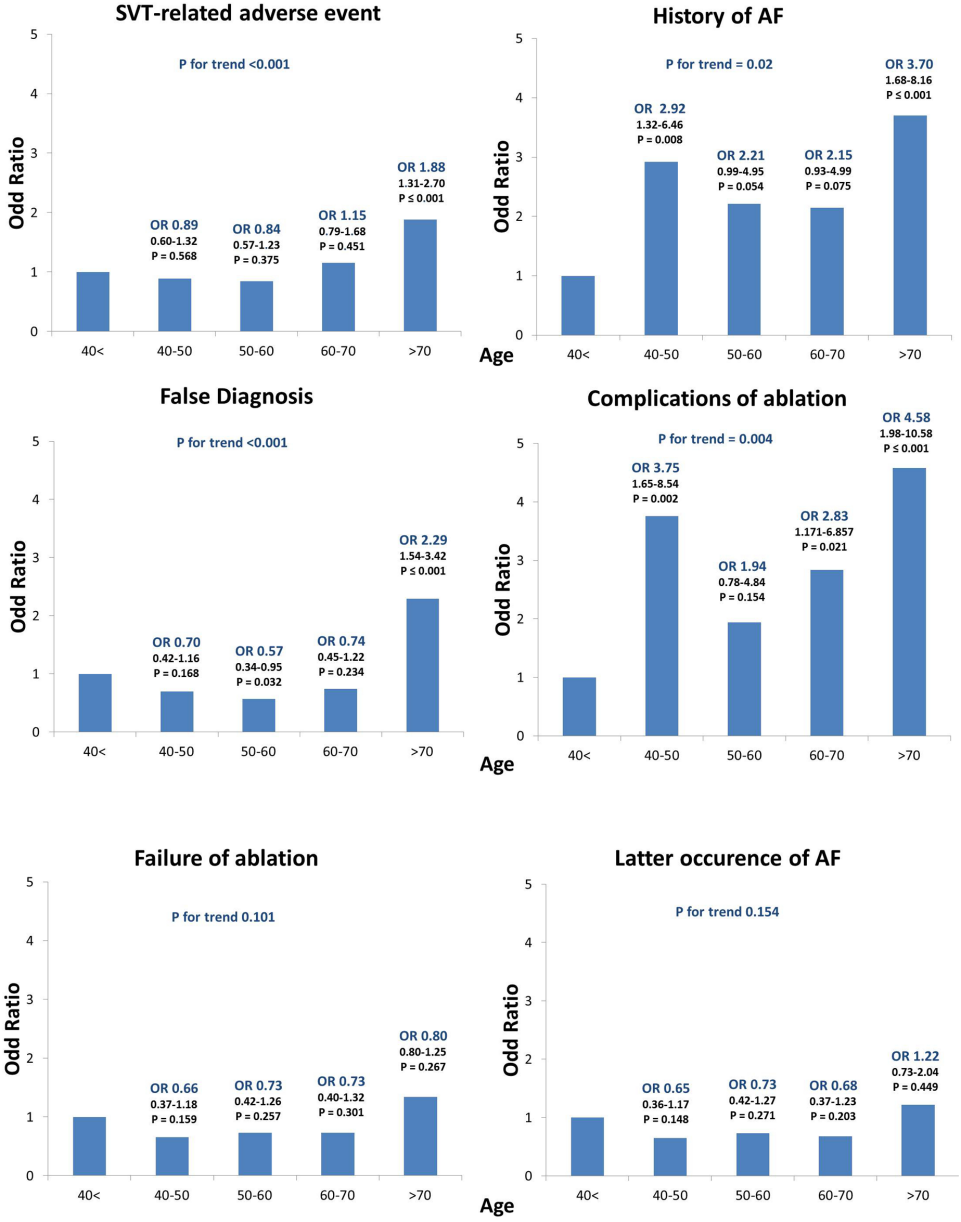
Adjusted association of age categories with SVT-related adverse events, history of AF, false diagnosis, complications of ablation, ablation failure and AF during follow-up as outcome. Adjustments were performed as in Table 4.

To further refine our assessment of the association between age and both clinical presentation and outcome, the <70 group was subdivided into 4 age groups (<40, n = 642; 40 to 50, n = 334; 50 to 60, n = 368 and 60 to 70, n = 315). Using the same adjustment as in Table 4, the adjusted association of age on all outcomes was calculated, taking the <40 age category as reference group (Fig 3).

Overall, only patients >70 had a significantly higher risk of adverse events (OR for >70 = 1.89, 1.31–2.70, p < 0.001) and false diagnosis due to SVT prior to ablation (OR for >70 = 2.294, 1.54–3.42, p < 0.001), whereas the risk of AF history and complication of ablation increased as early as >40 years of age (for AF history, OR for 40 to 50 = 2.922, 1.32–6.46, p = 0.008; for complication of ablation, OR for 40 to 50 = 3.755, 1.65–8.54, p = 0.002) in comparison with patients <40 years.

No significant differences were found among age groups regarding failure of ablation or later occurrence of AF.

## Discussion

Much of the clinical and electrophysiological data collected in patients with proven SVT differed between patients <70 years and ≥70 years. Ablation was more frequently indicated in patients ≥70 years despite a more frequent occurrence of failure and complications. Later occurrence of stroke, pacemaker implantation and death were more frequent whereas recurrence of SVT was similar while false recurrences were less frequent in patients ≥70 years. In multivariate analysis, age ≥70 years was an independent factor for most outcomes (Table 4) with the exception of ablation failure. To the best of our knowledge, the present study represents the most comprehensive analysis of the impact of higher age in SVT patients in a large single-center cohort in addition to providing key clinical findings. Of special interest, our findings provide strong evidence of a sizeable proportion of erroneous diagnoses in elderly patients, which can lead to unnecessary and potentially dangerous treatment such as administration of antiarrhythmic and/or anticoagulant drugs.

### Notable differences in the elderly population compared with young and middle-age patients

As expected, clinical signs were often more critical with regard to syncope, heart failure and acute coronary syndromes. Poor tolerance in older patients has been recently reported in other studies [14]. This poor tolerance is probably explained by a higher rate of cardiac comorbidities such as associated heart disease. In addition, the risk of AF, stroke and death observed in elderly individuals during follow-up differs from the natural favorable prognosis of SVT reported in younger patients [15]. Our results are in keeping with the study published by Epstein et al. [16] which reported that SVT was usually well-tolerated in young patients, but was more likely associated with disabling symptoms and have a life-threatening potential in the elderly. In addition, antiarrhythmic agents are less well-tolerated and may be associated with a higher incidence of toxicity in the elderly [16], which would further increase the rate of symptoms observed in this population.

In the elderly, tachycardia recurrence has been reported to be more frequent [16], usually in patients with atypical clinical presentation and poor tolerance signs, which can involve vital prognosis [17]. In contrast, in the present cohort, we did not identify a higher risk of recurrence in elderly patients.

Of importance, the present results demonstrate that erroneous diagnosis of the mechanism of regular tachycardia recorded in patients ≥70 years is frequent, either because regular wide QRS complex tachycardias are mistakenly interpreted as ventricular tachycardia or regular narrow QRS complex tachycardias are mistakenly interpreted as atrial tachycardia. These erroneous diagnoses can lead to unnecessary and potentially harmful treatments including administering anticoagulants or antiarrhythmic drug. Anticoagulants have been advocated by key opinion leaders in patients with atrial tachycardia [4]. However, international guidelines do not specifically address this issue. Yet, in the current setting of high rates of misdiagnosis, the use of transesophageal electrophysiological study could be useful to establish the nature of the tachycardia [11] and, in certain cases, avoid unnecessary anticoagulant and/or antiarrhythmic treatment, all of which have been associated with harmful side effects.

### Ablation in the elderly population

The diagnosis of reentrant SVT typically leads to an indication of a curative treatment with radiofrequency ablation of the slow pathway or accessory pathway. RF ablation of AVNRT has previously been reported as highly effective and safe in patients ≥65 years of age despite a higher prevalence of structural heart disease, a higher incidence of preexisting prolonged PR intervals and longer AH intervals at baseline [6,7,16,18]. In addition, ablation has been reported to be associated with a greater improvement in quality of life in the elderly comparatively to younger patients [19]. However, previous studies reported that ablation must be performed cautiously in these older patients with left-sided accessory pathways [20]. In the present study, ablation failures were more frequent in patients ≥70 years, even after adjusting for potential confounders. Of note, ablation failure in the present cohort was frequently due to irreducible AF induced in patients with a history of AF. Importantly, despite the relatively high rate of ablation-related complications in elderly patients observed in previous as well as the present study, recommendations regarding the management of SVT have recently been issued [3] in which the use of catheter ablation of the accessory pathway was recommended as first line therapy in patients with AF and/or AVRT. **Of note, the risk of procedural complications of ablation increased significantly as early as >40 years old in our population.** This finding is somewhat reassuring since it clearly indicates that the procedural risk does not exponentially increase with age. Nonetheless, it is possible that the older individuals in our referral cohort were highly selected, and consequently experiencing lower adverse procedural outcomes than their counterparts who were not referred for SVT workup. In addition, other outcomes such as pacemaker implantation and death are expected with older age.

### AF in the elderly population with supra-ventricular tachycardia

In the present cohort, the proportion of patients with a history of AF was significantly increased as early as >40 years while age was not associated with later occurrence of AF. SVT ablation did not appear to be protective of AF occurrence in patients with previously documented AF. History of AF and increased atrial vulnerability were moreover found to be risk factors of later AF. Arimoto et al. [21] also reported that physicians should consider regular long-term follow-up of older patients with atrial vulnerability in order to assess the subsequent development of AF. Importantly, in patients without AF history or AF during follow-up in the present series, 17 patients (1.1%) in those aged <70 and 9 (3.4%) in those aged 70 or older experienced a stroke during follow-up (Table 3). This could either indicate that patients with SVT have a noteworthy risk of stroke irrespective of the presence of AF or that AF is under-diagnosed during the follow-up of these patients. Nonetheless, further studies are warranted to ascertain whether SVT is associated *per se* with a higher risk of stroke compared to the risk observed in a matched sample of the general population.

### Clinical implications

Patients with SVT are usually young patients. However, in this particular setting and as demonstrated in other fields of medicine [22], older patients may be under-referred to specialized care. Invasive electrophysiological evaluation is mainly recommended in asymptomatic subjects before the age of 40 years in the case of preexcitation syndrome [23,24] as well as in patients with documented tachycardias, although no age thresholds have been proposed, along with no proposed age limits in the evaluation of SVT. Notwithstanding the latter, as we recently reported for other arrhythmias [9,11], the overall results of the present study would suggest that there is no definitive barrier in referring older patients with supraventricular tachycardia and that, consequently, such referral to specialized care may ultimately be favored. This is further emphasized by the high misdiagnosis rate observed in the present cohort of elderly patients. Transesophageal electrophysiological study may hence be of valuable interest in order to achieve a better diagnosis, and thus limit the risk of complications. Indeed, avoiding these erroneous diagnoses can prevent unnecessary and potentially dangerous treatment with anticoagulant and/or antiarrhythmic drugs, thereby suggesting the putative value of SVT diagnosis confirmed by electrophysiological study in elderly populations.

However, given the higher risk of ablation complications observed in elderly patients herein, along with the results of previous reports [18,25], the present findings would argue for a tempered recommendation for ablation in the elderly in which the procedural risks should be discussed with the patient prior to the intervention. Nevertheless, in the present cohort, only patients <40 years had a markedly lower risk for procedural complication, whereas patients 40–50 and >70 years had a similar risk of complications in multivariable analysis (OR = 3.76, 1.65–8.54 and OR = 4.58, 1.98–10.58 respectively), which would argue against limiting the access of patients >70 years solely on the basis of procedural risk. However, a risk-to-benefit ratio evaluation appears of critical importance in these patients. These results necessitate further validation in other large cohorts including prospective studies in order to better assess the overall risk-to-benefit ratio in the elderly population.

### Study limitations

This study suffers from all the limitations of observational single-center cohorts. Nevertheless, the homogeneity of patient management due to this single-center setting likely increases its internal validity.

The study does not quantify the overall incidence of SVT misdiagnosis since it involves a referral cohort–and consequently carries a degree of selection bias. In addition, misdiagnosis without EPS is probably preventable by increasing the level of expertise and of clinical investigation devoted to diagnosis identification, although this issue is beyond the scope of the present analysis. Importantly, access to a cardiologist in France is relatively straightforward, France having one of the highest ratio of cardiologists per inhabitant among Western countries [26]. Lastly, EPS also has its caveats, including a certain degree of enduring uncertainty despite its use, since the latter may fail to detect the true cause of tachycardia symptoms. In addition, invasive EPS is associated with a moderate risk of complication, which should trigger a careful benefit-risk evaluation in elderly patients. Importantly, our study does not show any impact of EPS on patient’s prognosis; such impact could be assessed only in clinical trials.

## Conclusions

A sizeable proportion of patients with supraventricular tachycardia without pre-excitation syndromes are old. In elderly patients with regular narrow-QRS tachycardia, an erroneous first diagnosis of atrial tachycardia derived from surface ECG can occur in a sizable number of cases and can lead to unnecessary treatments including potentially harmful interventions in some patients (antiarrhythmic and/or anticoagulant treatment) thereby supporting the performing of transesophageal EPS in most patients and invasive EPS in selected patients. The similar risk of SVT recurrence in elderly patients observed after ablation would further support an invasive strategy, in spite of the higher risk of failure and/or complication of ablation. Yet, invasive EPS and ablation does carry a risk of complication including severe ones. In these patients, a careful and extensive risk-to-benefit ratio assessment appears of critical importance. The present data could help in better assessing this risk-to-benefit ratio according to age in a given patient.

## References

[1] Furberg CD, Psaty BM, Manolio TA, Gardin JM, Smith VE, Rautaharju PM. Prevalence of atrial fibrillation in elderly subjects (the Cardiovascular Health Study). Am J Cardiol. 1994; 74: 236–41. 8037127 10.1016/0002-9149(94)90363-8

[2] Kirchhof P, Benussi S, Kotecha D, Ahlsson A, Atar D, Casadei B, et al 2016 ESC Guidelines for the management of atrial fibrillation developed in collaboration with EACTS. Eur Heart J. 2016; 37: 2893–962. doi: 10.1093/eurheartj/ehw210 27567408 10.1093/eurheartj/ehw210

[3] Page RL, Joglar JA, Caldwell MA, Calkins H, Conti JB, Deal BJ, et al 2015 ACC/AHA/HRS Guideline for the Management of Adult Patients With Supraventricular Tachycardia: A Report of the American College of Cardiology/American Heart Association Task Force on Clinical Practice Guidelines and the Heart Rhythm Society. J Am Coll Cardiol. 2016; 67: e27–e115. doi: 10.1016/j.jacc.2015.08.856 26409259 10.1016/j.jacc.2015.08.856

[4] Patel A, Markowitz SM. Atrial tachycardia: mechanisms and management. Expert Rev Cardiovasc Ther. 2008; 6: 811–22. doi: 10.1586/14779072.6.6.811 18570619 10.1586/14779072.6.6.811

[5] Orejarena LA, Vidaillet H Jr., DeStefano F, Nordstrom DL, Vierkant RA, Smith PN, et al Paroxysmal supraventricular tachycardia in the general population. J Am Coll Cardiol. 1998; 31: 150–7. 9426034 10.1016/s0735-1097(97)00422-1

[6] Meiltz A, Zimmermann M. Atrioventricular nodal reentrant tachycardia in the elderly: efficacy and safety of radiofrequency catheter ablation. Pacing Clin Electrophysiol. 2007; 30 Suppl 1: S103–7. 17302682 10.1111/j.1540-8159.2007.00616.x

[7] Rostock T, Risius T, Ventura R, Klemm HU, Weiss C, Keitel A, et al Efficacy and safety of radiofrequency catheter ablation of atrioventricular nodal reentrant tachycardia in the elderly. J Cardiovasc Electrophysiol. 2005; 16: 608–10. doi: 10.1111/j.1540-8167.2005.40717.x 15946358 10.1111/j.1540-8167.2005.40717.x

[8] Yangni N'Da O, Brembilla-Perrot B. Clinical characteristics and management of paroxysmal junctional tachycardia in the elderly. Arch Cardiovasc Dis. 2008; 101: 143–8. 18477940 10.1016/s1875-2136(08)71795-9

[9] Brembilla-Perrot B, Sellal JM, Olivier A, Manenti V, Villemin T, Beurrier D, et al Risk and outcome after ablation of isthmus-dependent atrial flutter in elderly patients. PLoS One. 2015; 10: e0127672 doi: 10.1371/journal.pone.0127672 26000772 10.1371/journal.pone.0127672 PMC4441372

[10] Brembilla-Perrot B, Girerd N, Sellal JM, Olivier A, Manenti V, Villemin T, et al Risk of atrial fibrillation after atrial flutter ablation: impact of AF history, gender, and antiarrhythmic drug medication. J Cardiovasc Electrophysiol. 2014; 25: 813–20. doi: 10.1111/jce.12413 24654647 10.1111/jce.12413

[11] Brembilla-Perrot B, Olivier A, Sellal JM, Manenti V, Brembilla A, Villemin T, et al Influence of advancing age on clinical presentation, treatment efficacy and safety, and long-term outcome of pre-excitation syndromes: a retrospective cohort study of 961 patients included over a 25-year period. BMJ Open. 2016; 6: e010520 doi: 10.1136/bmjopen-2015-010520 27188807 10.1136/bmjopen-2015-010520 PMC4874160

[12] Khachab H, Brembilla-Perrot B. Prevalence of atrial fibrillation in patients with history of paroxysmal supraventricular tachycardia. Int J Cardiol. 2013; 166: 221–4. doi: 10.1016/j.ijcard.2011.10.091 22078397 10.1016/j.ijcard.2011.10.091

[13] Brembilla-Perrot B, Sellal JM, Olivier A, Manenti V, Beurrier D, de Chillou C, et al Recurrences of symptoms after AV node re-entrant tachycardia ablation: a clinical arrhythmia risk score to assess putative underlying cause. Int J Cardiol. 2015; 179: 292–6. doi: 10.1016/j.ijcard.2014.11.071 25464467 10.1016/j.ijcard.2014.11.071

[14] Brembilla-Perrot B, Benichou M, Brembilla A, Bozec E, Dorlet S, Sellal JM, et al AV nodal reentrant tachycardia or AV reentrant tachycardia using a concealed bypass tract-related adverse events. Int J Cardiol. 2015; 199: 84–9. doi: 10.1016/j.ijcard.2015.07.048 26188825 10.1016/j.ijcard.2015.07.048

[15] D'Este D, Zoppo F, Bertaglia E, Zerbo F, Picciolo A, Scarabeo V, et al Long-term outcome of patients with atrioventricular node reentrant tachycardia. Int J Cardiol. 2007; 115: 350–3. doi: 10.1016/j.ijcard.2006.04.035 16814416 10.1016/j.ijcard.2006.04.035

[16] Epstein LM, Chiesa N, Wong MN, Lee RJ, Griffin JC, Scheinman MM, et al Radiofrequency catheter ablation in the treatment of supraventricular tachycardia in the elderly. J Am Coll Cardiol. 1994; 23: 1356–62. 8176093 10.1016/0735-1097(94)90377-8

[17] Wang YS, Scheinman MM, Chien WW, Cohen TJ, Lesh MD, Griffin JC. Patients with supraventricular tachycardia presenting with aborted sudden death: incidence, mechanism and long-term follow-up. J Am Coll Cardiol. 1991; 18: 1711–9. 1960318 10.1016/0735-1097(91)90508-7

[18] Zado ES, Callans DJ, Gottlieb CD, Kutalek SP, Wilbur SL, Samuels FL, et al Efficacy and safety of catheter ablation in octogenarians. J Am Coll Cardiol. 2000; 35: 458–62. 10676694 10.1016/s0735-1097(99)00544-6

[19] Bathina MN, Mickelsen S, Brooks C, Jaramillo J, Hepton T, Kusumoto FM. Radiofrequency catheter ablation versus medical therapy for initial treatment of supraventricular tachycardia and its impact on quality of life and healthcare costs. Am J Cardiol. 1998; 82: 589–93. 9732885 10.1016/s0002-9149(98)00416-0

[20] Chen SA, Chiang CE, Yang CJ, Cheng CC, Wu TJ, Wang SP, et al Accessory pathway and atrioventricular node reentrant tachycardia in elderly patients: clinical features, electrophysiologic characteristics and results of radiofrequency ablation. J Am Coll Cardiol. 1994; 23: 702–8. 8113555 10.1016/0735-1097(94)90757-9

[21] Arimoto T, Watanabe T, Nitobe J, Iwayama T, Kutsuzawa D, Miyamoto T, et al Difference of clinical course after catheter ablation of atrioventricular nodal reentrant tachycardia between younger and older patients: atrial vulnerability predicts new onset of atrial fibrillation. Intern Med. 2011; 50: 1649–55. 21841321 10.2169/internalmedicine.50.5280

[22] Blank L, Baird W, Reuber M. Patient perceptions of the referral of older adults to an epilepsy clinic: do patients and professionals agree who should be referred to a specialist? Epilepsy Behav. 2014; 34: 120–3. doi: 10.1016/j.yebeh.2014.03.019 24739450 10.1016/j.yebeh.2014.03.019

[23] Pappone C, Santinelli V, Rosanio S, Vicedomini G, Nardi S, Pappone A, et al Usefulness of invasive electrophysiologic testing to stratify the risk of arrhythmic events in asymptomatic patients with Wolff-Parkinson-White pattern: results from a large prospective long-term follow-up study. J Am Coll Cardiol. 2003; 41: 239–44. 12535816 10.1016/s0735-1097(02)02706-7

[24] Pediatric, Congenital Electrophysiology S, Heart Rhythm S, American College of Cardiology F, American Heart A, American Academy of P, et al PACES/HRS expert consensus statement on the management of the asymptomatic young patient with a Wolff-Parkinson-White (WPW, ventricular preexcitation) electrocardiographic pattern: developed in partnership between the Pediatric and Congenital Electrophysiology Society (PACES) and the Heart Rhythm Society (HRS). Endorsed by the governing bodies of PACES, HRS, the American College of Cardiology Foundation (ACCF), the American Heart Association (AHA), the American Academy of Pediatrics (AAP), and the Canadian Heart Rhythm Society (CHRS). Heart Rhythm. 2012; 9: 1006–24. doi: 10.1016/j.hrthm.2012.03.050 22579340 10.1016/j.hrthm.2012.03.050

[25] Kennedy R, Oral H. Catheter ablation of atrial fibrillation in the elderly: does the benefit outweigh the risk? Expert Rev Cardiovasc Ther. 2013; 11: 697–704. doi: 10.1586/erc.13.2 23750679 10.1586/erc.13.2

[26] Brembilla-Perrot B, Girerd N, Sellal JM, Olivier A, Manenti V et al Risk of atrial fibrillation after atrial flutter ablation: impact of AF history, gender, and antiarrhythmic drug medication. J Cardiovasc Electrophysiol 2014; 25: 813–2 doi: 10.1111/jce.12413 24654647 10.1111/jce.12413

